# Cancer patients with clonal hematopoiesis die from primary malignancy or comorbidities despite higher rates of transformation to myeloid neoplasms

**DOI:** 10.1002/cam4.7093

**Published:** 2024-03-18

**Authors:** Kelly S. Chien, Faustine Ong, Kunhwa Kim, Ziyi Li, Rashmi Kanagal‐Shamanna, Courtney D. DiNardo, Koichi Takahashi, Guillermo Montalban‐Bravo, Danielle Hammond, Koji Sasaki, Sherry A. Pierce, Hagop M. Kantarjian, Guillermo Garcia‐Manero

**Affiliations:** ^1^ Department of Leukemia The University of Texas MD Anderson Cancer Center Houston Texas USA; ^2^ Department of Biostatistics The University of Texas MD Anderson Cancer Center Houston Texas USA; ^3^ Department of Hematopathology The University of Texas MD Anderson Cancer Center Houston Texas USA

**Keywords:** clonal hematopoiesis, clonal hematopoiesis of indeterminate potential, clonal cytopenia of undetermined significance, CHIP, CCUS

## Abstract

**Background:**

The occurrence of somatic mutations in patients with no evidence of hematological disorders is called clonal hematopoiesis (CH). CH, whose subtypes include CH of indeterminate potential and clonal cytopenia of undetermined significance, has been associated with both hematologic cancers and systemic comorbidities. However, CH's effect on patients, especially those with concomitant malignancies, is not fully understood.

**Methods:**

We performed a retrospective evaluation of all patients with CH at a tertiary cancer center. Patient characteristics, mutational data, and outcomes were collected and analyzed.

**Results:**

Of 78 individuals included, 59 (76%) had a history of cancer and 60 (77%) had moderate to severe comorbidity burdens. *DNMT3A*, *TET2*, *TP53*, and *ASXL1* were the most common mutations. For the entire cohort, the 2‐year overall survival rate was 79% (95% CI: 70, 90), while the median survival was not reached. Of 20 observed deaths, most were related to primary malignancies (*n* = 7, 35%), comorbidities (*n* = 4, 20%), or myeloid neoplasms (*n* = 4, 20%). Twelve patients (15%) experienced transformation to a myeloid neoplasm. According to the clonal hematopoiesis risk score, the 3‐year transformation rate was 0% in low‐risk, 15% in intermediate‐risk (*p* = 0.098), and 28% in high‐risk (*p* = 0.05) patients. By multivariate analysis, transformation was associated with variant allele frequency ≥0.2 and hemoglobin <10 g/dL.

**Conclusions:**

In a population including mostly cancer patients, CH was associated with comorbidities and myeloid transformation in patients with higher mutational burdens and anemia. Nevertheless, such patients were less likely to die of their myeloid neoplasm than of primary malignancy or comorbidities.

## Graphical abstract

In a population including mostly cancer patients, clonal hematopoiesis was associated with comorbidities and myeloid transformation in patients with higher mutational burdens and anemia. Nevertheless, such patients were less likely to die of their myeloid neoplasm than of primary malignancy or comorbiditie**s**.

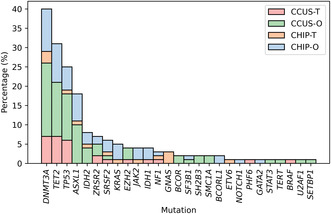

## 1 INTRODUCTION

The presence of somatic mutations in a subpopulation of hematopoietic cells in individuals without a morphological diagnosis is known as clonal hematopoiesis (CH). CH includes patients with CH of indeterminate potential (CHIP) and those with clonal cytopenia of undetermined significance (CCUS).^1^ Several studies have shown that CH is associated with comorbidities, such as cardiovascular disease, and with a predisposition to develop secondary hematologic malignancies.^2^, ^3^, ^4^, ^5^ In addition, the presence of pre‐leukemic CH prior to cancer therapy is common in patients, who later develop therapy‐related myeloid disorders,^6^, ^7^, ^8^, ^9^ a phenomenon likely related to increased fitness of the underlying CH.^10^ However, no guidelines exist for hematologic monitoring, comorbidity mitigation, and leukemia prevention in patients with CH. Furthermore, the impact of CH on the outcomes of patients with concomitant underlying malignancies remains unknown. To gain insight into the natural history of CH in this patient population, we analyzed the characteristics and outcomes of a cohort of patients with CH from a single tertiary cancer center.

## 2 METHODS

### 2.1 Study design and participants

In this retrospective analysis, we evaluated the characteristics and outcomes of a cohort of patients with CHIP and CCUS actively being observed at The University of Texas MD Anderson Cancer Center. Included were patients in whom one or more somatic mutations was found by next‐generation sequencing (NGS) of their bone marrow or peripheral blood from January 2015 through March 2021. CHIP was defined as the presence of CH with no cytopenia or morphologic findings of hematologic malignancy, while CCUS was defined as the presence of CH with cytopenia, using the established criteria of persistent anemia (hemoglobin <11 g/dL); neutropenia (absolute neutrophil count <1.5 K/μL); or thrombocytopenia (platelets <100 K/μL) for at least 4 months.^11^, ^12^ Excluded were patients, who had morphologic evidence of a myeloid neoplasm, previous history of any myeloid neoplasm, and/or donor‐engrafted CHIP. Patients' comorbidities were assessed according to the validated Adult Comorbidity Evaluation 27 (ACE‐27) score.^13^, ^14^

### 2.2 Assessment of somatic mutations

Genomic DNA from patients' peripheral blood and/or whole bone marrow aspirate samples was sequenced with an NGS platform in a Clinical Laboratory Improvement Amendments (CLIA)‐certified laboratory within the Department of Hematopathology at The University of Texas MD Anderson Cancer Center.^15^, ^16^

### 2.3 Outcome definitions and statistical methods

The date of CH diagnosis was defined as the date of the first identification of any somatic mutations. The date of transformation to myelodysplastic syndrome (MDS), chronic myelomonocytic leukemia (CMML), or acute myeloid leukemia (AML) was derived from the date of the first bone marrow evaluation showing morphological evidence of a myeloid malignancy. Overall survival (OS) was calculated from the time of CH diagnosis to the time of death. OS after transformation was calculated from the time of MDS/AML diagnosis to the time of death. Time to transformation was calculated from the time of diagnosis to the time of transformation or death without transformation. The cumulative incidence of transformation was estimated with death without transformation as a competing event.

Among patient characteristics, comparisons between continuous variables were made using the Kruskal‐Wallis test, and comparisons between categorical variables were made using the *χ* ^2^ test. Paired two‐sample *t*‐test and Wilcoxon rank‐sum test were used to compare continuous variables before and after transformation to MDS/AML. The Kaplan–Meier product limit method and cumulative incidence function were used to estimate survival outcomes.^17^ The Cox proportional hazard method was used to analyze association between CH and time to hematologic malignancy transformation.^18^ The associations between CH and cumulative incidence of transformation to a myeloid neoplasm and death without such transformation were analyzed by competing risk regression (i.e., subdistributional hazard model).^19^ Hazard ratios (HR) and subdistribution hazard ratios (SHR) with 95% confidence intervals (CI) were reported for time‐to‐event analysis. All analyses were performed using GraphPad Prism 9.0.0 and RStudio Version 4.1.0.^20^

## 3 RESULTS

### 3.1 Patient characteristics and study cohort

We identified 78 patients with CH seen at our institution from January 2015 through March 2021. Forty‐five individuals (58%) were referred to our clinic due to an incidental finding of a myeloid somatic mutation in their bone marrow (*n* = 37, 48%) or peripheral blood sample (*n* = 10, 10%), while 33 (42%) were referred for an abnormal blood count. The patients' baseline characteristics are summarized in Table 1. The median age at CH diagnosis was 72 years (range: 41–95). Most patients were male (*n* = 46, 59%), had cardiovascular comorbidities (*n* = 57, 73%), and had a non‐myeloid cancer diagnosis (*n* = 59, 76%). The previous cancer diagnoses prior to CH were solid tumors (*n* = 36, 46%), lymphoma (*n* = 16, 21%), multiple myeloma (*n* = 3, 4%), and chronic lymphocytic leukemia (CLL) (*n* = 2, 3%).

**TABLE 1:** Baseline patients characteristics.

Characteristics	All Patients	CHIP‐O	CHIP‐T	CCUS‐O	CCUS‐T	*p*‐value
	*N* (%)/Median	*N* (%)/Median	*N* (%)/Median	*N* (%)/Median	*N* (%)/Median	
	[Range]	[Range]	[Range]	[Range]	[Range]	
	*n* = 78	*n* = 26	*n* = 6	*n* = 33	*n* = 13	
Age, years	72 [41–95]	72 [43–89]	66 [41–75]	72 [46–86]	72 [55–95]	0.36
Age ≥ 70	43 (55)	15 (58)	2 (33)	19 (58)	7 (54)	0.73
Male	46 (59)	14 (54)	3 (50)	20 (61)	9 (69)	0.78
ACE‐27 score						
0	2 (3)	1 (4)	0	1 (3)	0	0.23
1 (mild)	16 (21)	7 (27)	0	8 (24)	0	
2 (moderate)	23 (29)	8 (31)	2 (33)	11 (33)	2 (15)	
3 (severe)	37 (47)	10 (38)	4 (67)	13 (39)	11 (85)	
Comorbidities						
Oncologic	59 (76)	17 (65)	6 (100)	23 (70)	13 (100)	**0.04**
Cardiovascular	57 (73)	20 (77)	3 (50)	23 (70)	11 (85)	0.41
Psychiatric	19 (24)	7 (27)	2 (33)	4 (12)	6 (46)	0.09
Respiratory	19 (24)	7 (27)	2 (33)	9 (27)	1 (8)	0.48
Diabetes mellitus	16 (21)	4 (15)	0	9 (27)	3 (23)	
Neurologic	8 (10)	1 (4)	0	5 (15)	2 (15)	
Gastrointestinal	3 (4)	0	0	1 (3)	1 (8)	
Renal	3 (4)	1 (4)	0	2 (6)	0	
Obesity	2 (3)	0	0	2 (6)	0	
Rheumatologic	2 (3)	1 (4)	0	1 (3)	0	
Immunologic	1 (1)	0	0	1 (3)	0	
Substance abuse	1 (1)	0	0	0	1 (8)	
Number of prior cancers						
0	19 (24)	9 (35)	0	10 (30)	0	0.32
1	43 (55)	11 (42)	5 (83)	19 (58)	8 (62)	
2	12 (15)	4 (15)	1 (17)	4 (12)	3 (23)	
3	2 (3)	2 (8)	0	0	0	
4	2 (3)	0	0	0	2 (15)	
Prior treatment history						
Chemotherapy^a^	33 (43)	8 (31)	4 (67)	11 (33)	10 (77)	**0.02**
Alkylating agents	27 (35)	7 (27)	2 (33)	10 (30)	8 (61)	
Topoisomerase inhibitors	15 (19)	3 (11)	1 (17)	10 (30)	1 (8)	
Other	6 (8)	1 (4)	2 (33)	1 (3)	2 (15)	
Radiation therapy	28 (36)	7 (27)	4 (67)	8 (24)	9 (69)	**0.009**
Prior Autologous SCT	9 (12)	2 (8)	0	3 (9)	4 (31)	0.11
CAR‐T cell therapy	5 (6)	0	0	5 (15)	0	
None	34 (44)	16 (62)	0	16 (48)	2 (15)	**<0.001**
Laboratory values						
WBC (K/μL)	4.9 [0.7–32.6]	5.5 [2.8–15.4]	5.7 [4.9–12.1]	3.2 [1.3–32.6]	3.9 [0.7–10.4]	**0.0013**
ANC (K/μL)	2.8 [0.2–18.6]	3.3 [1.8–11.3]	3.9 [2.3–9.9]	1.6 [0.7–18.6]	1.9 [0.2–5.2]	**0.003**
Hb (g/dL)	11.3 [6.9–15.7]	13.4 [11.1–15.7]	12.7 [11.7–15.3]	10.1 [6.9–13.8]	9.6 [7.1–12.4]	**<0.0001**
MCV (fL)	95 [74–124]	93 [77–106]	91 [84–104]	97 [75–124]	96 [74–111]	0.35
Platelets (K/μL)	170.5 [9–521]	193.5 [91–521]	254 [116–291]	128 [9–425]	160 [22–318]	**0.02**
LDH (U/L)	222 [114–882]	216 [114–645]	234 [152–483]	233 [125–882]	190.5 [117–430]	0.59
BM blasts (%)	1 [0–4]	1 [0–4]	1.5 [0–3]	1 [0–4]	1 [0–2]	0.60
Cytogenetics^b^						
Diploid	55 (77)	20 (83)	5 (100)	23 (74)	7 (64)	0.22
Trisomy 8	1 (1)	1 (4)	0	0	0	
‐Y	5 (7)	0	0	4 (13)	1 (9)	
Complex karyotype	1 (1)	0	0	0	1 (9)	
Deletion 20q	2 (3)	1 (4)	0	0	1 (9)	
Other abnormalities	7 (10)	2 (8)	0	4 (13)	1 (9)	
Mutations^c^						
*DNMT3A*	29 (40)	8 (32)	2 (50)	14 (44)	5 (45)	0.76
*TP53*	20 (26)	5 (19)	1 (17)	9 (27)	5 (38)	0.58
*TET2*	22 (31)	7 (28)	0	10 (31)	5 (45)	0.58
*ASXL1*	13 (18)	5 (20)	1 (25)	7 (22)	0	0.97
*IDH2*	6 (8)	2 (8)	1 (17)	3 (9)	0	0.80
VAF	0.08 [0.01–0.73]	0.06 [0.01–0.49]	0.15 [0.02–0.34]	0.09 [0.01–0.50]	0.09 [0.01–0.73]	0.69
Presence of DTA mutation	51 (71)	16 (64)	3 (75)	23 (72)	9 (82)	0.74
Mutational pathways^c^						
Epigenetic modifier (EM)	59 (79)	18 (72)	4 (80)	27 (82)	10 (83)	0.80
Splicing factor (SF)	11 (17)	3 (13)	1 (25)	5 (18)	2 (18)	0.93
Transcription factor (TF)	3 (5)	2 (9)	1 (25)	0	0	0.08
Tumor suppressor (TS)	20 (28)	5 (21)	1 (25)	9 (30)	5 (38)	0.71
Signaling and kinase (SKP)	10 (14)	4 (16)	4 (67)	1 (3)	1 (9)	**<0.001**
CHRS risk category^d^						
High	21 (27)	4 (15)	1 (17)	11 (33)	5 (38)	0.24
Intermediate	38 (49)	12 (46)	3 (50)	17 (52)	6 (46)	
Low	16 (21)	9 (35)	2 (33)	3 (9)	2 (15)	

*Note*: **EM:** *ASXL1*, *TET2*, *DNMT3A*, *IDH1*, *IDH2*, *EZH2*, *BCOR*, *BCORL1*; **SF:** *SF3B1*, *SRSF2*, *U2AF1*, *ZRSR2*. **TF:** *ETV6*, *GATA2*, *NOTCH1*. **TS:** *TP53*, *PHF6*; **SKP**: *JAK2*, *KRAS*, *NF1*, *BRAF*, *STAT3*, *GNAS*.

The bolded values are the statistically significant values.

Abbreviations: ACE‐27, adult comorbidity evaluation 27; ANC, absolute neutrophil count; BM, bone marrow; CAR, chimeric antigen receptor; CCUS‐O, other clonal cytopenia of undetermined significance (CCUS); CCUS‐T, CCUS with causes of cytopenia; CHIP‐O, other clonal hematopoiesis of indeterminate potential (CHIP); CHIP‐T, CHIP with causes of cytopenia; DTA, *DNMT3A*, *TET2*, or *ASXL1*; Hb, hemoglobin; LDH, lactate dehydrogenase; MCV, median corpuscular volume; SCT, stem cell transplantation; VAF, variant allele frequency; WBC, white blood cell count.

^a^ Patients received single agent therapy or a combination of listed chemotherapies.

^b^ No cytogenetic data available for seven patients.

^c^ Percentages were corrected for the available data on each gene.

^d^ Unable to calculate in three patients.

**FIGURE 1:**
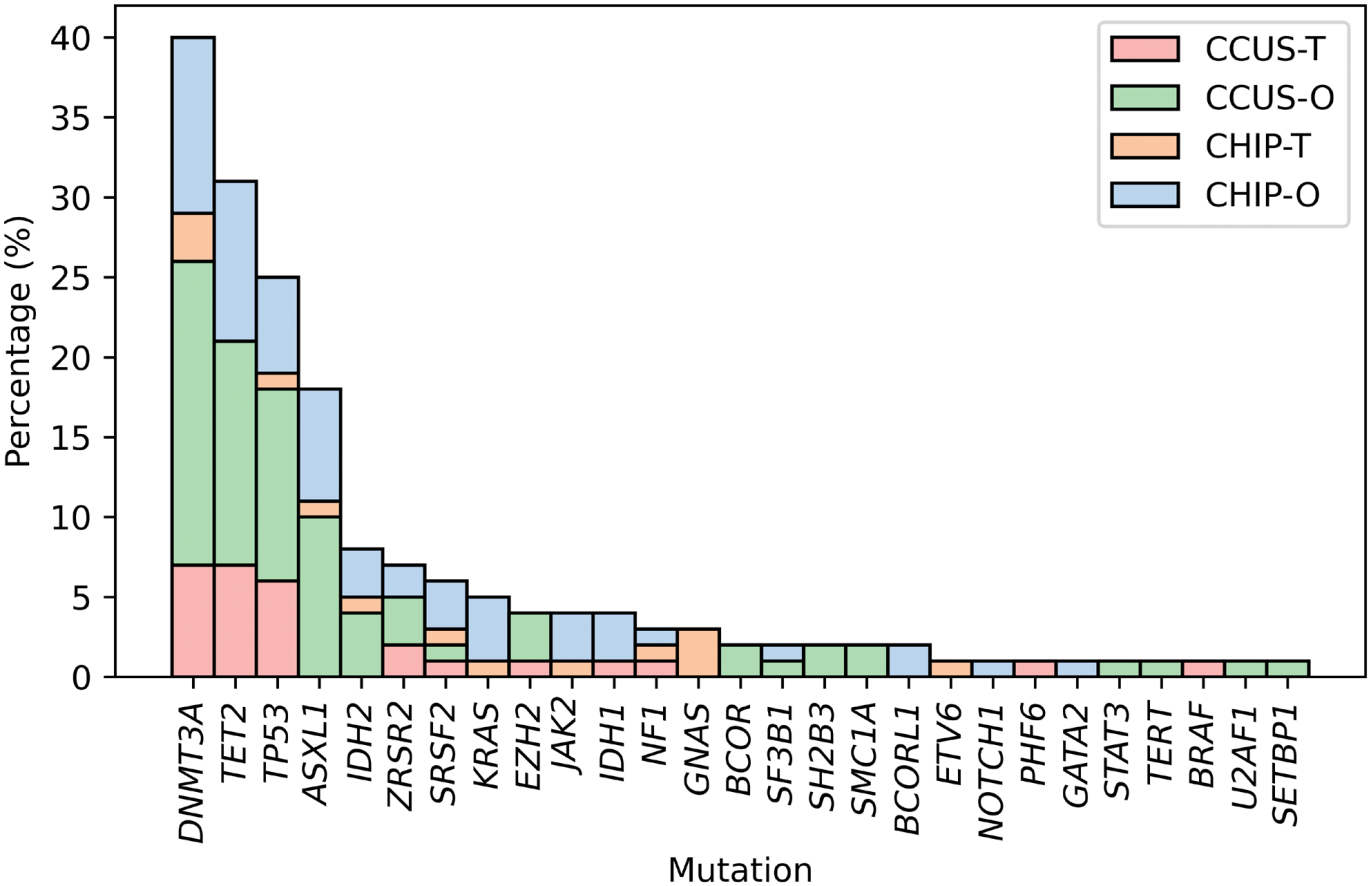
Distribution of mutations at diagnosis by cohort.

Genomic DNA was extracted from bone marrow samples in 70 patients (90%) and peripheral blood in eight individuals (9%). In those who were referred for a myeloid somatic mutation seen on peripheral blood, all underwent complete hematologic work‐up, including bone marrow evaluation, except four patients with no evidence of abnormalities on complete blood counts and differentials. The median number of mutations was one (range: 1–8), and the median variant allele frequency (VAF) was 0.078 (range: 0.011–0.729) at diagnosis. The distribution of somatic myeloid mutations is shown in Figure 1. *DNMT3A* (40%), *TET2* (31%), *TP53* (26%), and *ASXL1* (18%) were the most frequent mutations. Among the 71 patients with available cytogenetic data, 55 (77%) had normal karyotype and seven (10%) had a single chromosomal abnormality.

### 3.2 Establishment of subgroups by causes of cytopenia

Due to the possibility of other causes of cytopenia that cannot be distinguished from CH, such as treatment‐related myelosuppression or an active non‐myeloid malignancy (such as CLL or multiple myeloma), patients were further subcategorized. Of the 78 patients, 17 (22%) were receiving active treatment for a primary malignancy and two (3%) had active non‐myeloid hematologic malignancies without myelosuppressive therapy. Based on these potential causes for cytopenia, we categorized the patients into four cohorts: CHIP with normal counts in the setting of any cause for cytopenia (CHIP‐T), other CHIP (CHIP‐O), CCUS with any cause for cytopenia (CCUS‐T), and other CCUS (CCUS‐O) (Table 1). CHIP‐T patients tended to be younger, with a median age of 66 years (range: 41–75, *p* = 0.36). When we excluded primary malignancies from the ACE‐27 score,^13^, ^14^ there was a trend toward more CCUS‐T (*n* = 8, 62%) and CCUS‐O patients (*n* = 17, 52%) having moderate to severe comorbidities compared to only two CHIP‐T (33%) and 11 CHIP‐O (42%) individuals (*p* = 0.23). Regarding primary cancer therapy, more CCUS‐T patients received alkylating agents (*n* = 8, 61%, *p* = 0.02) with a trend toward more frequent autologous stem cell transplantation (SCT) (*n* = 4, 31%, *p* = 0.11). Five CCUS‐O patients (15%) received chimeric antigen receptor (CAR) T‐cell therapy for underlying lymphoma. Fifteen CHIP‐O (58%), 16 CCUS‐O (48%), and 2 CCUS‐T patients (15%) were never exposed to antineoplastic therapy prior to their CH diagnosis (*p* < 0.001). Bone marrow blast percentages were similar between the CHIP and CCUS cohorts with a median of 1% (range: 0–4, *p* = 0.60). Eight CCUS‐O (24%) and two CCUS‐T patients (15%) had transfusion‐dependent anemia at diagnosis.

No significant difference was seen in the number (*p* = 0.60) or median VAF (*p* = 0.69) of mutations across the groups. A trend of more frequent *TP53* mutations in CCUS‐T (*n* = 5, 38%) and CCUS‐O (*n* = 9, 27%) compared to CHIP‐T (*n* = 1, 17%) and CHIP‐O (*n* = 5, 19%) was observed (*p* = 0.58). However, CHIP‐T (*n* = 4, 67%) and CHIP‐O (*n* = 4, 16%) were more likely to have mutations in signaling and kinase pathway (SKP) genes compared to CCUS‐T (*n* = 1, 9%) and CCUS‐O (*n* = 1, 3%, *p* ≤ 0.001). Mutations in epigenetic modifiers (EM); splicing factor (SF) pathway genes; and *DNMT3A*, *TET2*, and/or *ASXL1* (DTA) were similar across all groups (*p* = 0.80, 0.93, and 0.74, respectively). No CCUS patient had mutations in transcription factor (TF) pathway genes, compared with 3 patients (9%) in the CHIP cohort.

### 3.3 Treatment

Twenty patients (26%), comprised of 16 CCUS‐O (48%) and 4 CCUS‐T (31%), received therapy for cytopenia, while all CHIP patients underwent observation. In those receiving treatment, approaches included growth factors (*n* = 15, 75%), such as erythropoiesis‐stimulating agents, granulocyte colony stimulating factor, and thrombopoietin‐stimulating agonists; corticosteroids (*n* = 5, 25%); cyclosporine (*n* = 5, 25%); iron supplementation (*n* = 4, 20%); intravenous immunoglobulin replacement (*n* = 3, 15%); rituximab (*n* = 2, 10%); and other non‐steroidal immunosuppressants (*n* = 1, 5%). Seven individuals (35%) received two or more therapies for refractory cytopenia. The median time to treatment from CH diagnosis was 1 month (range: 0–10).

### 3.4 Survival and transformation outcomes

With a median follow‐up time from diagnosis of 27 months (range: 0.1–79), the median OS (mOS) for the entire group was not reached, with a 2‐year survival rate of 79% (95% CI: 70, 90). The CCUS‐T cohort had the worst survival outcomes mOS: 32 months (95% CI: 32, not estimable [NE]), whereas the mOS of all other patients was not reached (*p* = 0.45, Figure S1). There were no differences in survival based by the presence of cytogenetic abnormalities (*p* = 0.970). Twenty patients (26%) died, of whom seven (35%) died of their primary cancer, four (20%) died of comorbidities, and four (20%) died of myeloid neoplasms following transformation (Table 2).

**TABLE 2:** Causes of death.

	All Patients *N* (%) *n* = 20	CHIP‐O *N* (%) *n* = 4	CHIP‐T *N* (%) *n* = 1	CCUS‐O *N* (%) *n* = 10	CCUS‐T *N* (%) *n* = 5
Primary malignancy	7 (35)	1 (25)	1 (100)	3 (30)	2 (40)
Comorbidities	4 (20)	1 (25)	0	1 (10)	2 (40)
Transformation to MN	4 (20)	1 (25)	0	3 (30)	0
Infection	3 (15)	0	0	3 (30)	0
Refractory anemia	1 (5)	0	0	0	1 (20)
Unknown	1 (5)	1 (25)	0	0	0

Abbreviations: CCUS‐O, other clonal cytopenia of undetermined significance (CCUS); CCUS‐T, CCUS with causes of cytopenia; CHIP‐O, other clonal hematopoiesis of indeterminate potential (CHIP); CHIP‐T, CHIP with causes of cytopenia; MN, myeloid neoplasm.

**FIGURE 2:**
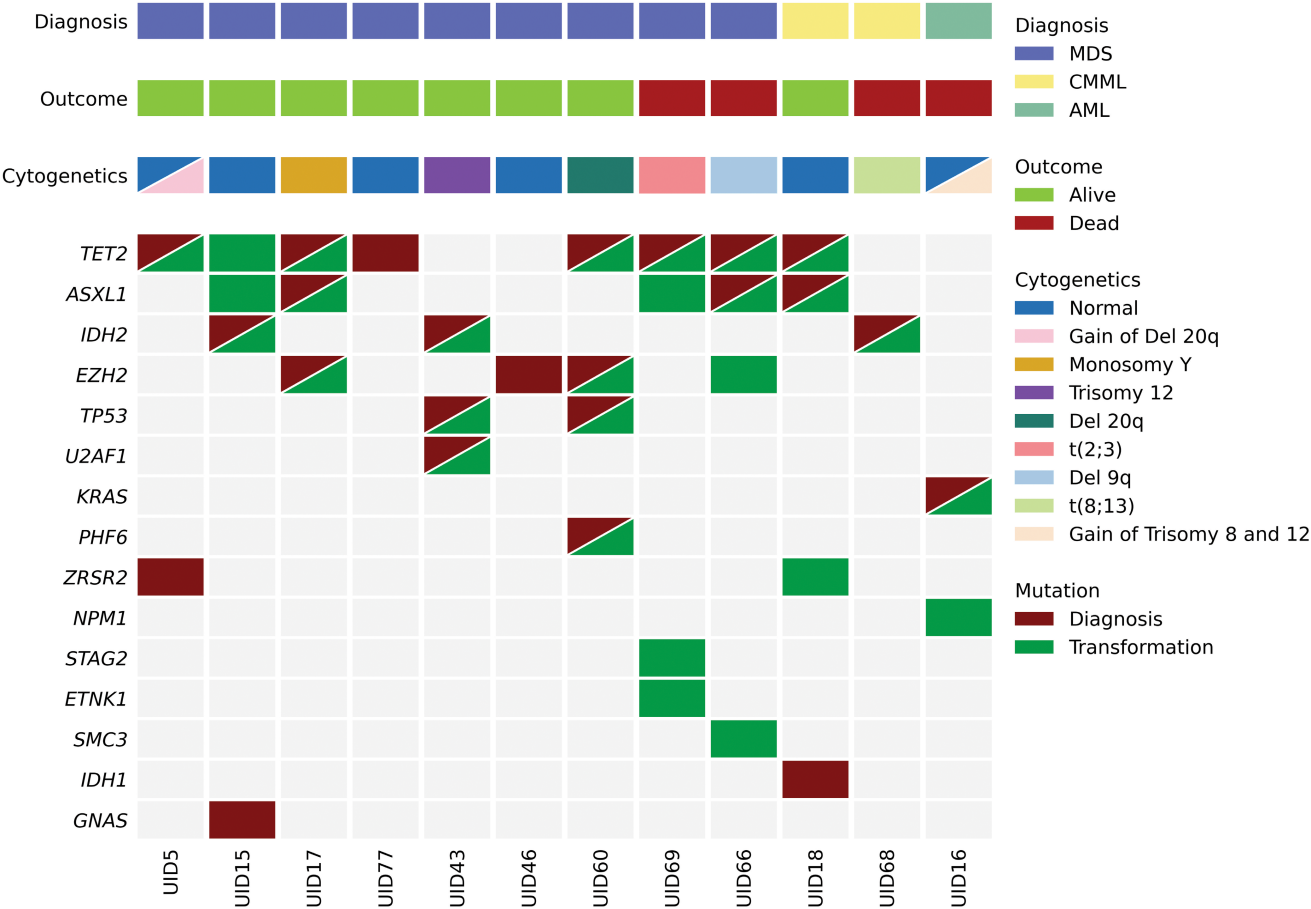
Oncoplot of Patients who transformed to MDS/CMML or AML. MDS, myelodysplastic syndrome; CMML, chronic myelomonocytic leukemia; AML, acute myeloid leukemia; UID, unique identifier.

Of the 78 patients, 12 (15%) experienced transformation to MDS/CMML (*n* = 11) or AML (*n* = 1). Patient characteristics and outcomes summarized in Table S1. Transformation occurred in seven CCUS‐O (21%), one CHIP‐T (17%), two CCUS‐T (15%), and two CHIP‐O patients (8%). The median time to transformation was not reached, and there was no significant difference in time to transformation among groups (*p* = 0.53, Figure S2). The mOS after transformation was also not reached. Of interest, the median number of mutations (two vs. three, *p* = 0.67) and VAF (0.36 vs. 0.37, *p* = 0.82) were similar at the time of CH diagnosis and at transformation, respectively. The most common mutations at transformation were *TET2* (64%), *ASXL1* (45%), *IDH2* (27%), *EZH2* (27%), and *TP53* (18%). All three patients (two CCUS‐O and one CCUS‐T) with *EZH2* mutations at baseline progressed to MDS. Figure 2 illustrates the genomic evolution from diagnosis to myeloid neoplasm transformation. Two patients (17%) acquired a new cytogenetic abnormality and five patients (42%) acquired new mutations at transformation, with *ASXL1* being the most frequently acquired mutation (40%). Six patients (50%) did not receive treatment for their lower‐risk MDS or CMML.

The cumulative incidence of transformation was further calculated with death without transformation as a competing event after combining the CHIP‐O and CHIP‐T subgroups due to small patient numbers. The 3‐year cumulative incidence of myeloid neoplasm transformation was higher among the CCUS‐O (24%) and CCUS‐T (21%) groups than in the CHIP group (6%, *p* = 0.48, Figure S3). CCUS‐T patients had the highest 3‐year cumulative incidence of death without transformation risk (53%) compared to the CCUS‐O (35%) and CHIP (20%, *p* = 0.14) groups, which reflected a higher proportion of deaths due to primary malignancy (40%) and comorbidities (40%) in the CCUS‐T group.

### 3.5 Exploratory analysis

**FIGURE 3:**
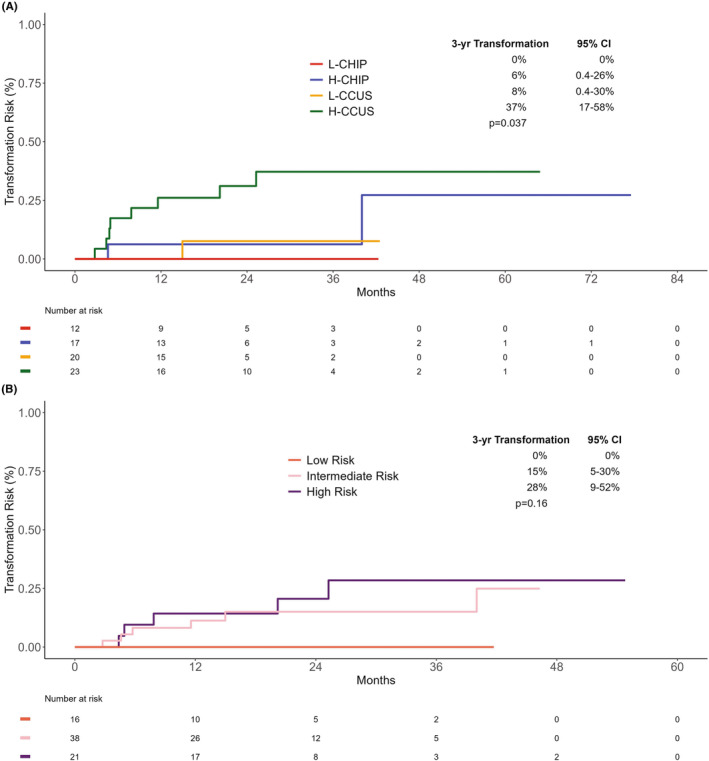
Cumulative incidence of transformation with risk stratification. (A) By VAF ≥0.2 and/or non‐DTA Mutations. CI, confidence interval; DTA, DNMT3A, TET2, or ASXL1; H‐CCUS, high‐risk CCUS; H‐CHIP, high‐risk CHIP; L‐CCUS, low‐risk clonal cytopenia of undetermined significance (CCUS); L‐CHIP, low‐risk clonal hematopoiesis of indeterminate potential (CHIP); VAF, variant allele frequency. (B) By Clonal Hematopoiesis Risk Score. CI, confidence interval.

Patients were further recategorized into high‐risk (H) or low‐risk (L) cohorts based on VAF ≥0.2 or non‐DTA mutations, which have been reported to portend a higher risk of progression.^21^ As illustrated in Figure 3A, the 3‐year cumulative incidence of myeloid neoplasm transformation was highest in the H‐CCUS group (37%) compared to the L‐CCUS (8%), H‐CHIP (6%), and L‐CHIP groups (0%, *p* = 0.037). Patients were also risk‐stratified according to the recently‐presented and published clonal hematopoiesis risk score (CHRS), a prediction tool for the risk of progression to myeloid neoplasms in healthy adults with CH.^22^ Per the CHRS, 16 patients (21%) were deemed at low risk, 38 (49%) at intermediate risk, and 21 (27%) at high risk of transformation to a myeloid neoplasm. There was no difference in CHRS risk group by previous exposure to chemotherapy, radiation therapy, or autologous stem cell transplantation. When examining particular chemotherapy regimens, alkylating agents were not enriched in a particular risk group (*p* = 0.380), but interestingly, no CHRS low‐risk individual received topoisomerase inhibitors prior to the detection of CHIP or CCUS (*p* = 0.077). CCUS‐T (38%) and CCUS‐O patients (33%) exhibited a trend toward a higher risk of myeloid neoplasm transformation than CHIP‐T (17%) and CHIP‐O (15%) patients, though the difference was not significant (*p* = 0.24). The 3‐year cumulative incidence of transformation was 0% in the low‐risk group compared to 15% in the intermediate‐risk (*p* = 0.098) and 28% in the high‐risk groups (*p* = 0.05, Figure 3B). There were no differences in time to transformation or mOS by CHRS risk stratification (Figure S4). When comparing the two risk stratification methods, of the 16 CHRS low‐risk patients, 15 were also categorized as L‐CHIP/L‐CCUS. One CHRS low‐risk patient was classified in the high‐risk cohort due to the presence of a mutation in *BCORL1* that is considered non‐DTA but not a high‐risk mutation by CHRS.

On univariate analysis, increasing age (HR 1.10; 95% CI: 1.02, 1.18; *p* = 0.008), VAF ≥0.2 (HR 5.75; 95% CI: 1.51, 21.8; *p* = 0.01), and hemoglobin <10 g/dL (HR 5.66; 95% CI: 1.51, 19.87; *p* = 0.007) were associated with higher risk of transformation (Table S2). DTA mutations were not statistically protective against transformation (HR 0.59; 95% CI: 0.16, 2.09; *p* = 0.41) in our cohort. Similar findings were obtained by competing risk analysis. Anemia with hemoglobin <10 g/dL was also associated with mortality without transformation risk (SHR 3.78; 95% CI: 1.30, 11.00; *p* = 0.01). On multivariate analysis, only VAF ≥0.2 (HR 15.62; 95% CI: 3.29, 74.05; *p* = 0.0005) and hemoglobin <10 g/dL (HR 18.25; 95% CI: 3.59, 92.73; *p* = 0.0005) predicted transformation (Table 3).

**TABLE 3:** Multivariate analysis of predictors of transformation.

	Full model^a^			Reduced model^b^		
	HR	95% CI	*p*‐value	HR	95% CI	*p*‐value
Age (years)	0.98	0.90–1.07	0.65			
Hb < 10 (g/dL)	21.85	2.45–194.83	0.006	18.25	3.59–92.73	0.0005
VAF ≥ 0.2	20.09	1.99–202.4	0.011	15.62	3.29–74.05	0.0005
Normal cytogenetics	0.88	0.10–7.78	0.91			

Abbreviations: CI, confidence interval; Hb, hemoglobin; HR, hazard ratio; VAF, variant allele frequency.

^a^ The full model includes variables with *p* < 0.15 on univariate analysis by Cox Regression.

^b^ The reduced model includes significant variables using backward elimination methods.

## 4 DISCUSSION

This retrospective study focused on 78 patients with CH, most of whom (76%) had a history of non‐myeloid neoplasms and exposure to chemotherapy and/or radiation. Patients in this cohort had high comorbidity burdens, with the majority of patients having moderate or severe ACE‐27 scores,^13^, ^14^ most frequently cardiovascular disorders in 73%. To determine patients who were at the highest risk of transforming into a myeloid malignancy, we further categorized patients into high‐risk and low‐risk cohorts based on VAF ≥0.2 and the presence of non‐DTA mutations,^21^ resulting in the H‐CCUS group with the highest cumulative incidence of transformation. We also applied CHRS to stratify patients according to their risk of transformation.^22^ Only VAF ≥0.2 and hemoglobin <10 g/dL predicted transformation to MDS/AML by multivariate analysis.

The patients at a tertiary cancer center differ from the healthy adult population, as they appear to have more high‐risk features. Two large population studies in the United Kingdom and the Netherlands showed low transformation rates, with development of myeloid neoplasms 2.37% over a median follow‐up time of almost 12 years and a cumulative incidence of a hematological cancer of 2.5% every 5 years.^22^, ^23^ However, the mutations most frequently seen in these studies were overwhelmingly *DNMT3A*, *TET2*, and *ASXL1*, with *TP53* mutations seen in approximately 2%–5% of the patients. Our study revealed *TP53* mutations in significantly more patients at 25%, indicating a higher‐risk cohort with consequently higher rates of transformation at 15%. Similar to these previous reports, *DNMT3A* mutations appear to portend an indolent CH course, as no patients with *DNMT3A* mutations transformed.

The impact of CH on therapy‐related myeloid neoplasms is an area of great interest. A recent study comparing 33 patients with CCUS following cytotoxic therapies to therapy‐related myeloid neoplasms found a transformation rate of 30%.^24^ Our study evaluating a different patient population resulted in lower transformation rates, though eight patients (67%) had a history of cancer and six (50%) received antineoplastic therapy. Shah et al. noted 44% of evaluable patients had clonal evolution at the time of transformation, similar to our observations.^24^ Another group of researchers conducted a multicenter CCUS data registry study evaluating those with a history of cancer, previous cytotoxic therapies, and all others.^25^ Though rates of transformation to myeloid neoplasms were not reported, patients who had received cytotoxic therapies had inferior mOS to those who did not. Our CCUS‐T group only incorporated those on active antineoplastic treatments but showed similar survival patterns. Interestingly, their study had an extremely low incidence of mutations in *TP53*, which was third‐most mutated in our patient cohort. Further identification of the cause of cytopenia in patients with CCUS, such as with CCUS‐T vs. CCUS‐O, are warranted for risk stratification.

Several studies have shown correlations of CH to comorbidities, including cardiac disease and autoimmune disorders.^4^, ^5^, ^26^ CH is associated with a high risk of atherosclerotic cardiovascular disease.^2^, ^4^, ^5^ Anti‐inflammatory agents, such as canakinumab in *TET2*‐mutated CH patients in the Canakinumab Anti‐Inflammatory Thrombosis Outcomes Study (CANTOS), may reduce CH‐related cardiovascular risk.^27^, ^28^ Other studies indicate that some types of CH may contribute to inflammatory conditions via T‐cell dysregulation and VEXAS syndrome, a potentially fatal autoimmune disorder with relapsing polychondritis.^26^, ^29^, ^30^ As 20% of deaths in our cohort were attributed to comorbidities, close monitoring of CH patients for extra‐hematologic manifestations is paramount.

The identification of CH patients who are at high risk of experiencing transformation to a myeloid neoplasm is another area of critical focus. In addition to the publication discussing VAF ≥0.2 and the presence of non‐DTA mutations,^21^ the CHRS was recently developed to identify individuals at risk of myeloid malignancies from CH.^22^ Prognostic variables include number of mutations, high risk mutations, VAF ≥0.2, age, cytopenia, and increased red blood cell indices by red cell distribution width and mean corpuscular volume. In our study investigating predominantly cancer patients, the CHRS was able to discern patients at low‐ versus intermediate‐risk and high‐risk of transformation (*p* = 0.098 and 0.05, respectively) but was less able to distinguish between intermediate‐ and high‐risk patients (*p* = 0.60). By multivariate analysis, only VAF ≥0.2 and hemoglobin <10 g/dL correlated with an elevated risk of transformation to myeloid malignancies. This finding highlights the need for further exploration to identify risk factors for progression to myeloid neoplasms in cancer patients. Specific antineoplastic agents, such as alkylating agents, topoisomerase inhibitors, and poly(ADP‐ribose) polymerase (PARP) inhibitors, have been implicated in the development of therapy‐related MDS/AML in some patients with CH.^10^, ^31^, ^32^, ^33^, ^34^ In our cohort, no low‐risk individual received topoisomerase inhibitors though there were no statistically significant differences in CHRS risk group by therapeutic subtypes. In those who eventually developed therapy‐related MDS/AML, CH has been discovered at the time of their primary cancer diagnosis, before they began antineoplastic therapy.^6^, ^7^ It is important that such patients be identified and engaged in discussions about the potential benefits and risks of anticancer treatments as well as possible modifications to therapy.

There are several limitations to our study. First, the follow‐up time is short at over 2 years. A prospective study with longer follow‐up times is needed to further explore these observations. Second, the number of patients included is small at 78 with much variability in baseline characteristics, and only 12 patients transformed to MDS or AML. Therefore, the statistical analysis has limited power in this situation. Moreover, the recently‐published World Health Organization (WHO) and International Consensus Classifications (ICC) of myeloid neoplasms require VAF ≥0.02 and higher cutoffs for cytopenia to distinguish between CHIP and CCUS.^35^, ^36^ We included the few patients with VAF <0.02 as many were diagnosed during the initial development of NGS; with the improvement of high‐throughput sequencing techniques, these individuals would likely have WHO/ICC‐defined CH with current technologies. We also used diagnostic criteria in MDS for cytopenia as this study was conducted prior to the development of WHO and ICC guidelines for CHIP and CCUS, and our patient population was sicker than that of healthy individuals on which most CH studies are based.^11^, ^12^ There were five patients who were recategorized from CHIP‐O to CCUS‐O by the new criteria with no difference in results. Additionally, we grouped patients into four cohorts based on cytopenia and the presence of an additional variable that could account for cytopenia, such as active non‐myeloid hematologic malignancies (multiple myeloma and chronic lymphocytic leukemia) or concurrent antineoplastic therapy. Due to paucity of laboratory values in treatment‐free intervals, we are unable to determine the fluctuation of blood counts and definitively associate cytotoxic therapy with cytopenia. Lastly, CH with *PPM1D* mutations has been implicated in therapy‐related myeloid neoplasms, but our myeloid NGS panel did not capture this mutation.

In conclusion, the mOS is not reached in cancer patients with CH after over 2 years of follow‐up. Progression to MDS/AML occurred in 15% of patients, but the primary causes of death were more frequently the primary malignancy and complication from another comorbid condition rather than transformation to MDS/AML. Patients with higher mutational burdens, represented by VAF ≥0.2, and anemia were at higher risk of transformation to a myeloid neoplasm. Both close monitoring of extra‐hematologic manifestations crucial and further investigation into identifying individuals at high risk of myeloid transformation and developing early therapeutic interventions for these patients are warranted.

## AUTHOR CONTRIBUTIONS

**Kelly S. Chien:** Conceptualization (equal); data curation (equal); formal analysis (equal); investigation (equal); methodology (equal); visualization (equal); writing – original draft (equal); writing – review and editing (equal). **Faustine Ong:** Conceptualization (equal); data curation (equal); formal analysis (equal); methodology (equal); writing – original draft (equal); writing – review and editing (equal). **Kunhwa Kim:** Conceptualization (equal); data curation (equal); formal analysis (equal); writing – review and editing (equal). **Ziyi Li:** Conceptualization (equal); formal analysis (equal); writing – review and editing (equal). **Rashmi Kanagal‐Shamanna:** Conceptualization (equal); data curation (equal); writing – review and editing (equal). **Courtney D. DiNardo:** Conceptualization (equal); supervision (equal); writing – original draft (equal); writing – review and editing (equal). **Koichi Takahashi:** Conceptualization (equal); supervision (equal); writing – original draft (equal); writing – review and editing (equal). **Guillermo Montalban‐Bravo:** Conceptualization (equal); writing – original draft (equal); writing – review and editing (equal). **Danielle Hammond:** Conceptualization (equal); writing – original draft (equal); writing – review and editing (equal). **Koji Sasaki:** Conceptualization (equal); writing – original draft (equal); writing – review and editing (equal). **Sherry Pierce:** Conceptualization (equal); data curation (equal); writing – original draft (equal); writing – review and editing (equal). **Hagop M. Kantarjian:** Conceptualization (equal); supervision (equal); writing – original draft (equal); writing – review and editing (equal). **Guillermo Garcia‐Manero:** Conceptualization (equal); formal analysis (equal); investigation (equal); methodology (equal); supervision (equal); writing – original draft (equal); writing – review and editing (equal).

## CONFLICT OF INTEREST STATEMENT

The authors have no relevant conflicts of interest to disclose.

## ETHICS STATEMENT

The study was approved by the Institutional Review Board at The University of Texas MD Anderson. A waiver of informed consent was granted.

## Supporting information


Table S1.

Figure S1.

Figure S2.

Figure S3.

Figure S4.

## Data Availability

Data available on request from the authors.

## References

[1] Steensma DP , Bejar R , Jaiswal S , et al. Clonal hematopoiesis of indeterminate potential and its distinction from myelodysplastic syndromes. Blood. 2015;126(1):9‐16. 25931582 10.1182/blood-2015-03-631747 PMC4624443

[2] Jaiswal S , Fontanillas P , Flannick J , et al. Age‐related clonal hematopoiesis associated with adverse outcomes. N Engl J Med. 2014;371(26):2488‐2498. 25426837 10.1056/NEJMoa1408617 PMC4306669

[3] Genovese G , Kahler AK , Handsaker RE , et al. Clonal hematopoiesis and blood‐cancer risk inferred from blood DNA sequence. N Engl J Med. 2014;371(26):2477‐2487. 25426838 10.1056/NEJMoa1409405 PMC4290021

[4] Jaiswal S , Natarajan P , Silver AJ , et al. Clonal hematopoiesis and risk of atherosclerotic cardiovascular disease. N Engl J Med. 2017;377(2):111‐121. 28636844 10.1056/NEJMoa1701719 PMC6717509

[5] Fuster JJ , MacLauchlan S , Zuriaga MA , et al. Clonal hematopoiesis associated with TET2 deficiency accelerates atherosclerosis development in mice. Science. 2017;355(6327):842‐847. 28104796 10.1126/science.aag1381 PMC5542057

[6] Takahashi K , Wang F , Kantarjian H , et al. Preleukaemic clonal haemopoiesis and risk of therapy‐related myeloid neoplasms: a case‐control study. Lancet Oncol. 2017;18(1):100‐111. 27923552 10.1016/S1470-2045(16)30626-X PMC5405697

[7] Gillis NK , Ball M , Zhang Q , et al. Clonal haemopoiesis and therapy‐related myeloid malignancies in elderly patients: a proof‐of‐concept, case‐control study. Lancet Oncol. 2017;18(1):112‐121. 27927582 10.1016/S1470-2045(16)30627-1 PMC7771361

[8] Coombs CC , Zehir A , Devlin SM , et al. Therapy‐related clonal hematopoiesis in patients with non‐hematologic cancers is common and associated with adverse clinical outcomes. Cell Stem Cell. 2017;21(3):374‐382.e374. 28803919 10.1016/j.stem.2017.07.010 PMC5591073

[9] Wong TN , Ramsingh G , Young AL , et al. Role of TP53 mutations in the origin and evolution of therapy‐related acute myeloid leukaemia. Nature. 2015;518(7540):552‐555. 25487151 10.1038/nature13968 PMC4403236

[10] Bolton KL , Ptashkin RN , Gao T , et al. Cancer therapy shapes the fitness landscape of clonal hematopoiesis. Nat Genet. 2020;52(11):1219‐1226. 33106634 10.1038/s41588-020-00710-0 PMC7891089

[11] Valent P , Horny HP , Bennett JM , et al. Definitions and standards in the diagnosis and treatment of the myelodysplastic syndromes: consensus statements and report from a working conference. Leuk Res. 2007;31(6):727‐736. 17257673 10.1016/j.leukres.2006.11.009

[12] Valent P . ICUS, IDUS, CHIP and CCUS: diagnostic criteria, separation from MDS and clinical implications. Pathobiology. 2019;86(1):30‐38. 29860246 10.1159/000489042 PMC7115849

[13] Piccirillo JF , Creech C , Zequeira R , Anderson S , Johnston AS . Inclusion of comorbidity into oncology data registries. J Registry Manag. 1999;26(2):66‐70.

[14] Piccirillo J , Costas I , Claybour P , Borah A , Grove L , Jeffe D . The measurement of comorbidity by cancer registries. J Registry Manag. 2003;30(1):8‐15.

[15] Kanagal‐Shamanna R , Luthra R , Yin CC , et al. Myeloid neoplasms with isolated isochromosome 17q demonstrate a high frequency of mutations in SETBP1, SRSF2, ASXL1 and NRAS. Oncotarget. 2016;7(12):14251‐14258. 26883102 10.18632/oncotarget.7350 PMC4924712

[16] Kanagal‐Shamanna R , Singh RR , Routbort MJ , Patel KP , Medeiros LJ , Luthra R . Principles of analytical validation of next‐generation sequencing based mutational analysis for hematologic neoplasms in a CLIA‐certified laboratory. Expert Rev Mol Diagn. 2016;16(4):461‐472. 26765348 10.1586/14737159.2016.1142374

[17] Aalen O , Borgan Ø , Gjessing H . Survival and event history analysis: a process point of view. 2008.

[18] Cox DR . Regression models and life‐tables. J R Stat Soc B Methodol. 1972;34(2):187‐202.

[19] Fine JP , Gray RJ . A proportional hazards model for the subdistribution of a competing risk. J Am Stat Assoc. 1999;94(446):496‐509.

[20] R: A Language and Environment for Statistical Computing [Computer Program]. R Foundation for Statistical Computing; 2022.

[21] He R , Chiou J , Chiou A , et al. Molecular markers demonstrate diagnostic and prognostic value in the evaluation of myelodysplastic syndromes in cytopenia patients. Blood Cancer J. 2022;12(1):12. 35078969 10.1038/s41408-022-00612-w PMC8789920

[22] Weeks LD , Niroula A , Neuberg D , et al. Prediction of risk for myeloid malignancy in clonal hematopoiesis. NEJM Evid. 2023;2(5):eVIDoa2200310. 10.1056/evidoa2200310 PMC10361696 37483562

[23] van Zeventer IA , de Graaf AO , Salzbrunn JB , et al. Evolutionary landscape of clonal hematopoiesis in 3,359 individuals from the general population. Cancer Cell. 2023;41(6):1017‐1031. 37146604 10.1016/j.ccell.2023.04.006

[24] Shah MV , Mangaonkar AA , Begna KH , et al. Therapy‐related clonal cytopenia as a precursor to therapy‐related myeloid neoplasms. Blood Cancer J. 2022;12(7):106. 35803921 10.1038/s41408-022-00703-8 PMC9270475

[25] Xie Z , Smith A , Komrokji RS , et al. The characteristics and prognosis of patients with clonal cytopenias of undetermined significance, including cancer and therapy‐related clonal cytopenias. Blood. 2022;140(Supplement 1):2887‐2890.

[26] Sikora KA , Wells KV , Bolek EC , Jones AI , Grayson PC . Somatic mutations in rheumatological diseases: VEXAS syndrome and beyond. Rheumatology (Oxford). 2022;61(8):3149‐3160. 34888629 10.1093/rheumatology/keab868 PMC9348615

[27] Ridker PM , Everett BM , Thuren T , et al. Antiinflammatory therapy with canakinumab for atherosclerotic disease. N Engl J Med. 2017;377(12):1119‐1131. 28845751 10.1056/NEJMoa1707914

[28] Svensson EC , Madar A , Campbell CD , et al. TET2‐driven clonal hematopoiesis and response to canakinumab: an exploratory analysis of the CANTOS randomized clinical trial. JAMA Cardiol. 2022;7(5):521‐528. 35385050 10.1001/jamacardio.2022.0386 PMC8988022

[29] Kusne Y , Fernandez J , Patnaik MM . Clonal hematopoiesis and VEXAS syndrome: survival of the fittest clones? Semin Hematol. 2021;58(4):226‐229. 34802544 10.1053/j.seminhematol.2021.10.004

[30] Zhao L‐P , Boy M , Azoulay C , et al. MDS/CMML with TET2 or IDH mutation are associated with systemic inflammatory and autoimmune diseases (SIAD) and T cell dysregulation. Blood. 2020;136:31‐32.

[31] Ganser A , Heuser M . Therapy‐related myeloid neoplasms. Curr Opin Hematol. 2017;24(2):152‐158. 27930389 10.1097/MOH.0000000000000316

[32] Sperling AS , Guerra VA , Kennedy JA , et al. Lenalidomide promotes the development of TP53‐mutated therapy‐related myeloid neoplasms. Blood. 2022;140(16):1753‐1763. 35512188 10.1182/blood.2021014956 PMC9837415

[33] Morice P‐M , Leary A , Dolladille C , et al. Myelodysplastic syndrome and acute myeloid leukaemia in patients treated with PARP inhibitors: a safety meta‐analysis of randomised controlled trials and a retrospective study of the WHO pharmacovigilance database. Lancet Haematol. 2021;8(2):e122‐e134. 33347814 10.1016/S2352-3026(20)30360-4

[34] Almanza‐Huante E , Bataller A , Urrutia S , et al. Outcomes of patients with therapy‐related myeloid neoplasms after treatment with poly(ADP‐ribose) polymerase proteins inhibitors for solid tumours. Br J Haematol. 2023;201(3):e25‐e29. 36951293 10.1111/bjh.18766

[35] Khoury JD , Solary E , Abla O , et al. The 5th edition of the World Health Organization classification of Haematolymphoid tumours: myeloid and histiocytic/dendritic neoplasms. Leukemia. 2022;36(7):1703‐1719. 35732831 10.1038/s41375-022-01613-1 PMC9252913

[36] Arber DA , Orazi A , Hasserjian RP , et al. International consensus classification of myeloid neoplasms and acute leukemias: integrating morphologic, clinical, and genomic data. Blood. 2022;140(11):1200‐1228. 35767897 10.1182/blood.2022015850 PMC9479031

