# Supplementary material for: Cancer patients with clonal hematopoiesis die from primary malignancy or comorbidities despite higher rates of transformation to myeloid neoplasms
Source: Cancer Med. 2024 Mar 18;13(5):e7093. doi: 10.1002/cam4.7093 (PMC10945882; doi:10.1002/cam4.7093)

**Supplemental Table 1.** Characteristics of Patients who Transformed to MDS/CMML or AML.

| **Pt #** | **Age** | **Gender** | **Cohort**  **(Cytopenia Present)** | **At Diagnosis** | | **Type of Myeloid Neoplasm** | **At Transformation** | | **MDS/AML Treatment** | **Time to Transformation (months)** | **Dead** | **Follow-Up (months)** |
| --- | --- | --- | --- | --- | --- | --- | --- | --- | --- | --- | --- | --- |
|  |  |  |  | **CG** | **NGS (VAF%)** |  | **CG** | **NGS (VAF%)** |  |  |  |  |
| 5 | 75 | M | CCUS-T  (Neutropenia) | Normal | *TET2* (72.9), *ZRSR2* (52.5) | MDS | del(20q) | *TET2* (90.4) | observation | 25.3 | no | 39.92 |
| 15 | 42 | F | CHIP-T | Normal | *GNAS* (0.5), *IDH2* (19.7) | MDS | Normal | *ASXL1* (5.6), *IDH2* (26.1), *TET2* (11.1) | observation | 5.75 | no | 24.02 |
| 16 | 73 | F | CHIP-O | Normal | *KRAS* (36.2) | AML | +8, +12 | *KRAS* (44), *NPM1* (15.6) | cladribine + low-dose cytarabine alternating with decitabine | 4.60 | yes | 7.13 |
| 17 | 79 | M | CCUS-O  (Neutropenia) | -Y | *ASXL1* (19.1), *EZH2* (20.4), *TET2* x2 (37.7, 40.7) | MDS | -Y | *ASXL1* (14.1), *EZH2* (21.7), *TET2* x2 (41.2, 41.8) | observation | 11.56 | no | 34.23 |
| 18 | 78 | M | CHIP-O | Normal | *ASXL1* (37.7), *IDH1* (49.4), *TET2* x2 (31.9, 42.5) | CMML | Normal | *ASXL1* (37.5), *TET2* x2 (43.9, 44.7), *ZRSR2* (3.3) | observation | 40.02 | no | 78.88 |
| 43 | 72 | M | CCUS-O  (Anemia) | Normal | *IDH2* (12.5), *TP53* (1.9), *U2AF1* (12) | MDS | Normal | *IDH2* (12.5), *U2AF1* (10.3) | decitabine | 20.20 | no | 43.50 |
| 46 | 69 | F | CCUS-O (Anemia, Thrombocytopenia) | Normal | *EZH2* (1.3%) | MDS | Normal | None | azacitidine with no response 🡪 Treg with transient benefit 🡪 observation | 2.76 | no | 32.49 |
| 60 | 80 | M | CCUS-T  (Anemia) | del(20q) | *EZH2* (1.3), *PHF6* x2 (5.8, 12.2), *TET2* (38), *TP53* (1.1) | MDS | del(20q) | *TET2* (40.7), *TP53* (1.3), *EZH2* (3.7), *PHF6* x2 (10.1, 14.4) | observation | 4.37 | no | 13.11 |
| 66 | 86 | M | CCUS-O  (Anemia) | del(9q) | *ASXL1* (43.4), *TET2* (49.5) | MDS | del(9q) | *ASXL1* (31.4), *EZH2* (15.8), *TET2* (11.3), *SMC3* (4.4) | lenalidomide + ESA 🡪 ESA alone | 7.85 | yes | 24.71 |
| 68 | 86 | M | CCUS-O  (Anemia, Thrombocytopenia) | t(8;13) | *IDH2* (25.6) | CMML | t(8;13) | *IDH2* (25.6) | observation | 4.93 | yes | 8.71 |
| 69 | 86 | F | CCUS-O  (Neutropenia, Anemia) | t(2;3) | *TET2* (42.1%) | MDS | t(2;3) | *STAG2* (27.6), *TET2* x2 (34.3, 36.1), *ASXL1* (17.7), *ETNK1* (32.5) | azacitidine + ipilimumab | 4.80 | yes | 7.10 |
| 77 | 71 | F | CCUS-O  (Anemia, Thrombocytopenia) | Normal | *TET2* (8.8) | MDS | Normal | Not performed | decitabine + G-CSF | 14.98 | no | 51.48 |

MDS: myelodysplastic syndrome; CMML: chronic myelomonocytic leukemia; AML: acute myeloid leukemia; CG: conventional cytogenetics; NGS: next-generation sequencing mutational panel; VAF: variant allele frequency; M: male; F: female; ESA: erythropoiesis-stimulating agent; G-CSF: granulocyte colony stimulating factor

**Supplemental Table 2.** Univariate Analysis of Predictors for Transformation.

| Variables | Risk of Transformation by Cox Regression | | | Competing Risk Analysis | | | | | |
| --- | --- | --- | --- | --- | --- | --- | --- | --- | --- |
|  |  |  |  | Risk of Transformation | | | Risk of Mortality without Transformation | | |
|  | HR | 95% CI | P-value | SHR | 95% CI | P-value | SHR | 95% CI | P-value |
| Age (year) | 1.10 | 1.02-1.18 | **0.008** | 1.07 | 1.02-1.13 | **0.0059** | 1.05 | 0.99-1.11 | 0.12 |
| Male | 1.10 | 0.32-3.78 | 0.87 | 0.971 | 0.285-3.32 | 0.96 | 3.44 | 0.82-14.4 | 0.09 |
| Prior malignancies | 0.52 | 0.15-1.78 | 0.30 | 0.561 | 0.165-1.91 | 0.35 | 1.09 | 0.303-3.93 | 0.89 |
| Presence of DTA mutation | 0.59 | 0.16-2.09 | 0.41 | 0.67 | 0.20-2.23 | 0.51 | 0.61 | 0.21-1.76 | 0.36 |
| VAF ≥ 0.2 | 5.75 | 1.51-21.8 | **0.01** | 5.79 | 1.55-21.7 | **0.009** | 0.63 | 0.19-2.13 | 0.46 |
| ≥2 mutations | 1.36 | 0.42-4.47 | 0.61 | 1.39 | 0.425-4.55 | 0.59 | 0.72 | 0.24-2.15 | 0.55 |
| Diploid cytogenetics | 0.38 | 0.11-1.24 | 0.11 | 0.34 | 0.102-1.14 | 0.079 | 4.23 | 0.55-32.6 | 0.17 |
| ANC < 1.5 (K/µL) | 0.65 | 0.14-3.13 | 0.60 | 0.75 | 0.17-3.28 | 0.70 | 0.44 | 0.11-1.77 | 0.25 |
| Hb < 10 (g/dL) | 5.66 | 1.61-19.87 | **0.007** | 4.26 | 1.3-13.9 | **0.017** | 3.78 | 1.3-11 | **0.01** |
| Platelet < 100 (K/µL) | 1.24 | 0.33-4.69 | 0.75 | 1.23 | 0.31-4.93 | 0.77 | 1.72 | 0.66-4.5 | 0.27 |
| EM mutations | 2.65 | 0.33-20.96 | 0.36 | 2.87 | 0.36-23 | 0.32 | 0.56 | 0.19-1.66 | 0.29 |
| SF mutations | 2.33 | 0.45-12.1 | 0.31 | 2.11 | 0.47-9.49 | 0.33 | 0.96 | 0.12-5.01 | 0.96 |
| TS mutations | 0.76 | 0.16-3.69 | 0.74 | 1.1 | 0.22-5.58 | 0.91 | 0.80 | 0.24-2.64 | 0.70 |
| SKP mutations | 2.25 | 0.26-19.58 | 0.46 | 1.7 | 0.21-13.6 | 0.62 | 2.75 | 0.60-12.7 | 0.19 |

HR: hazard ratio; CI: confidence interval; SHR: subdistribution hazard ratio; DTA: *DNMT3A*, *TET2*, or *ASXL1*; VAF: variant allele frequency; ANC: absolute neutrophil count; Hb: hemoglobin; EM: epigenetic modifier; SF: splicing factor; TS: tumor suppressor; SKP: signaling and kinase pathway

**Supplemental Figure 1.** Overall Survival by Cohort.


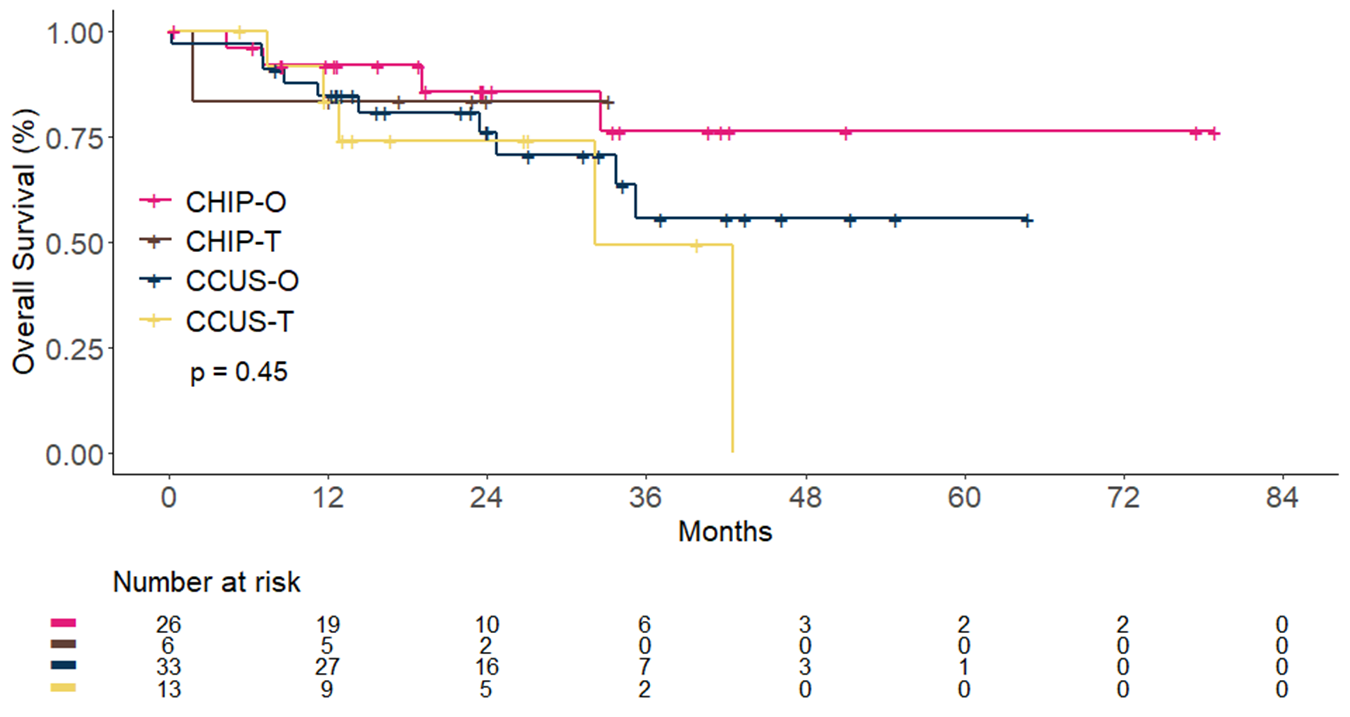


CHIP-O: other clonal hematopoiesis of indeterminate potential (CHIP); CHIP-T: CHIP with causes of cytopenia; CCUS-O: other clonal cytopenia of undetermined significance (CCUS); CCUS-T: CCUS with causes of cytopenia

**Supplemental Figure 2.** Time to Transformation by Cohort.


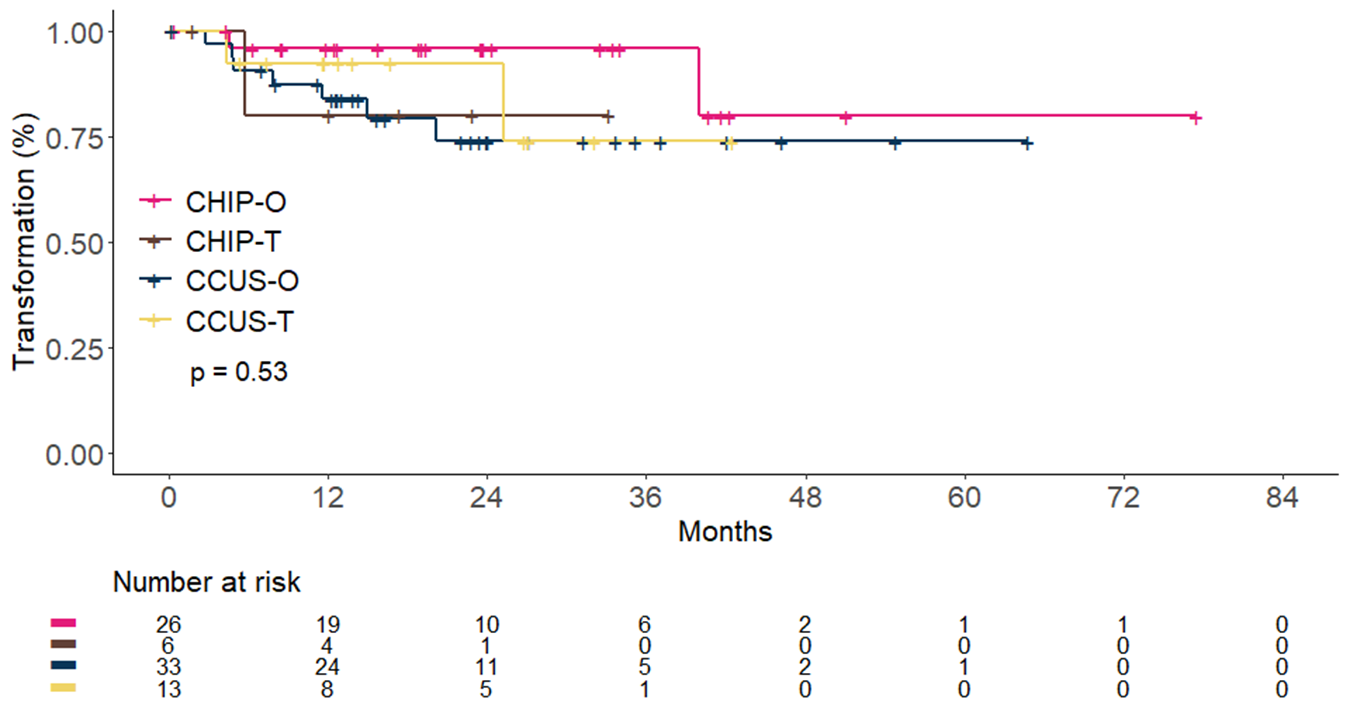


CHIP-O: other clonal hematopoiesis of indeterminate potential (CHIP); CHIP-T: CHIP with causes of cytopenia; CCUS-O: other clonal cytopenia of undetermined significance (CCUS); CCUS-T: CCUS with causes of cytopenia

**Supplemental Figure 3.** Cumulative Incidence of Transformation by Cohort.


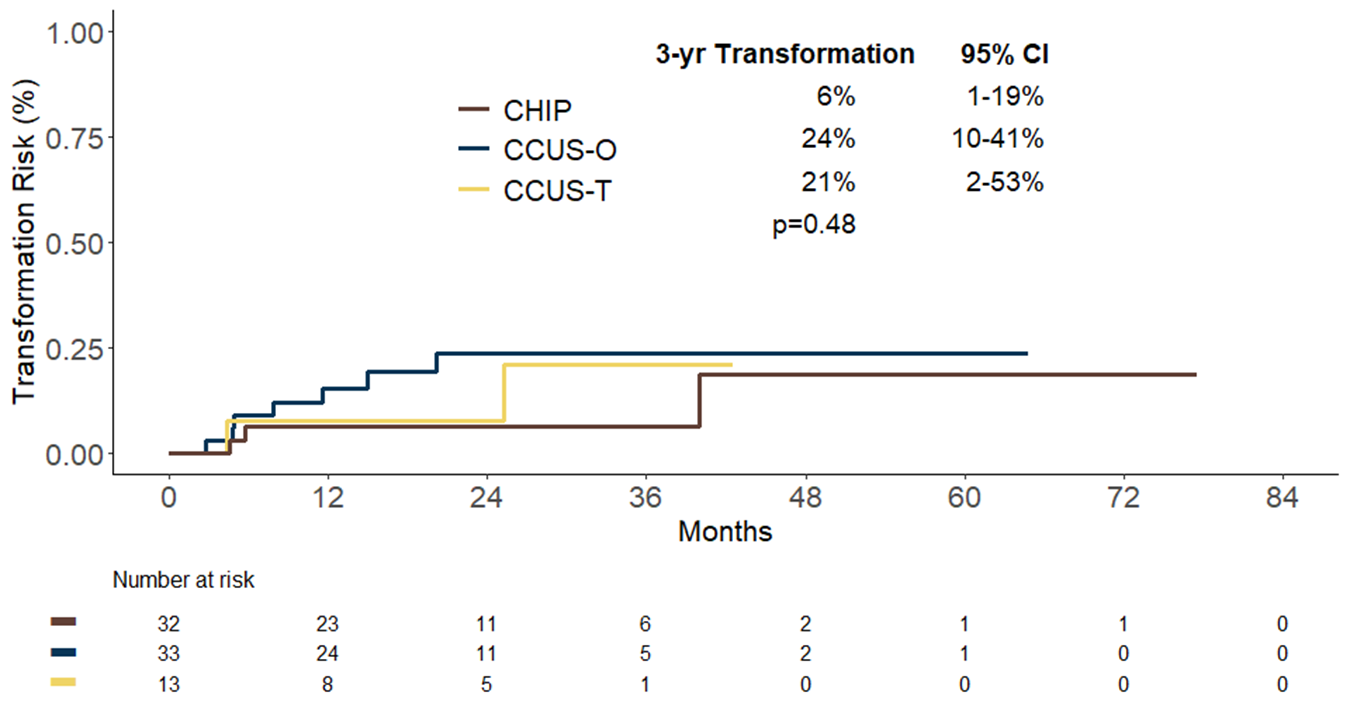


CHIP: clonal hematopoiesis of indeterminate potential (CHIP); CCUS-O: other clonal cytopenia of undetermined significance (CCUS); CCUS-T: CCUS with causes of cytopenia

**Supplemental Figure 4.** Outcomes by Clonal Hematopoiesis Risk Stratification Risk Group.

1. Time to Transformation.


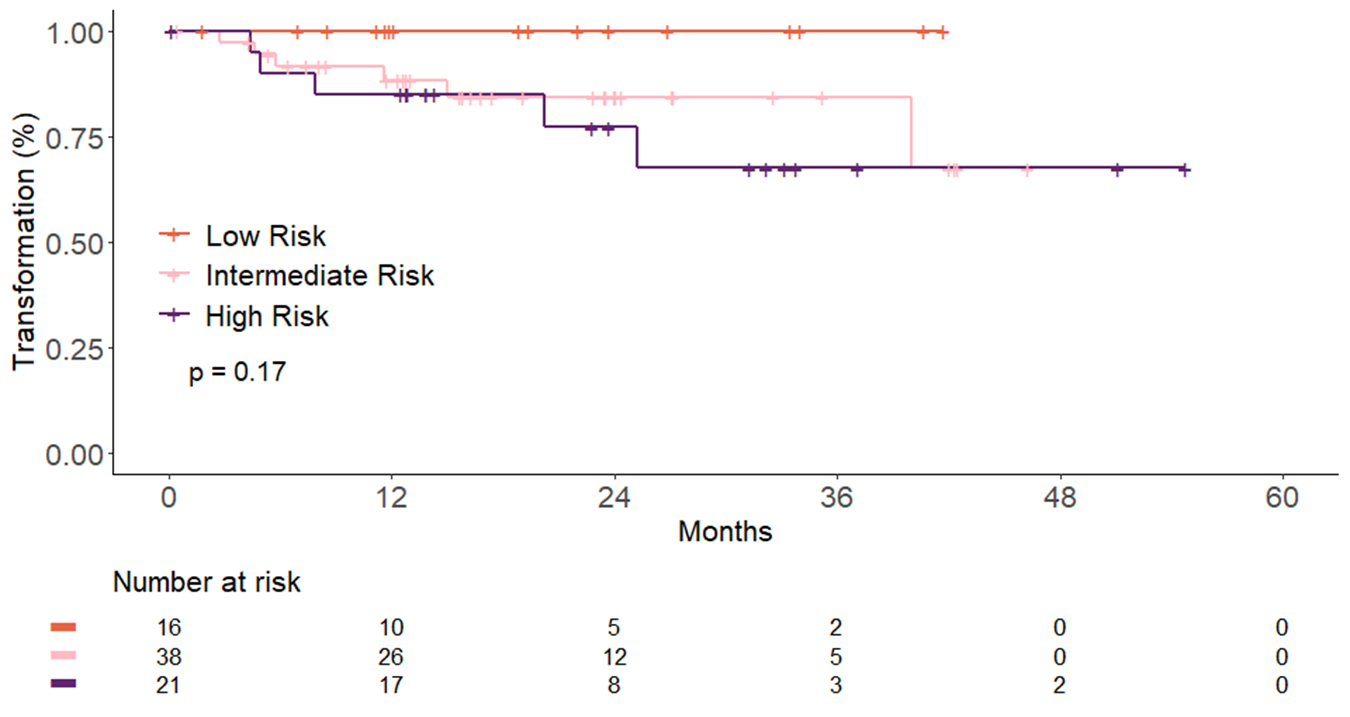


1. Overall Survival.


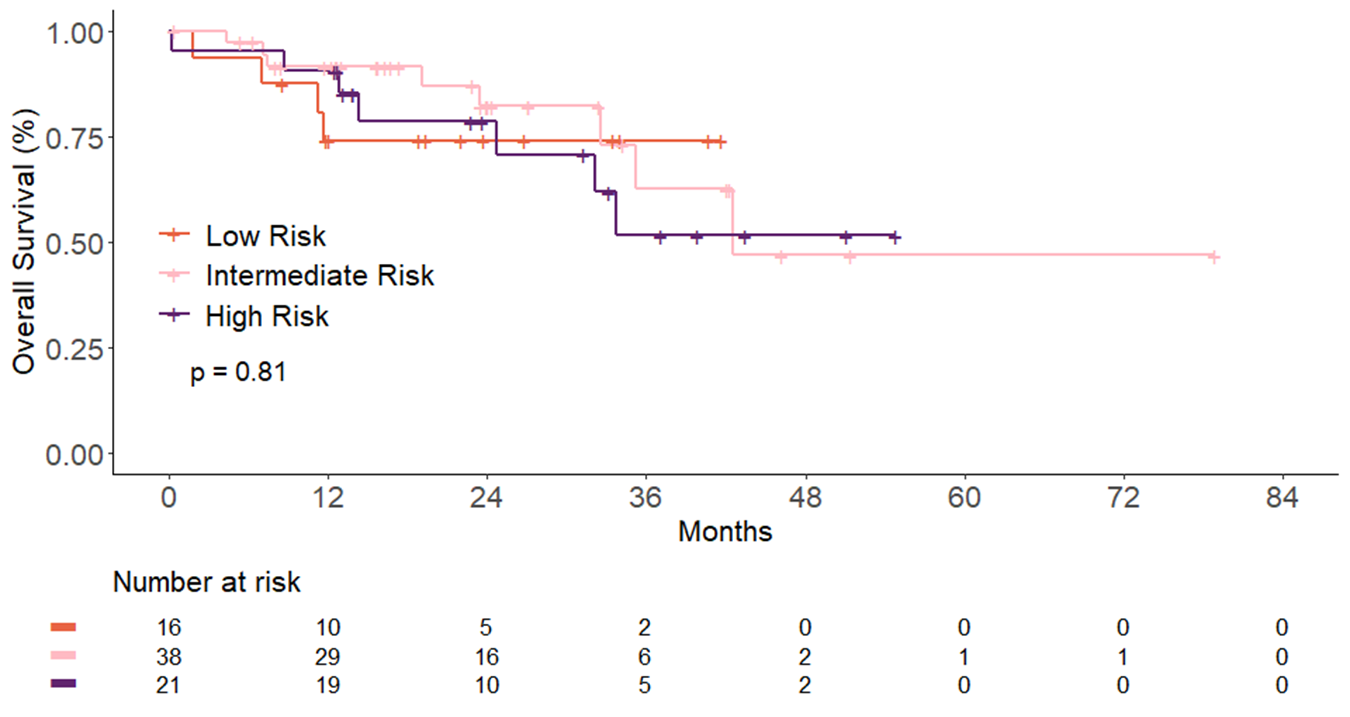

Supplement: Supplementary file 1 — Table S1. [file CAM4-13-e7093-s001.docx]
