# Supplementary figures and images for: Cancer patients with clonal hematopoiesis die from primary malignancy or comorbidities despite higher rates of transformation to myeloid neoplasms
Source: Cancer Med. 2024 Mar 18;13(5):e7093. doi: 10.1002/cam4.7093 (PMC10945882; doi:10.1002/cam4.7093)

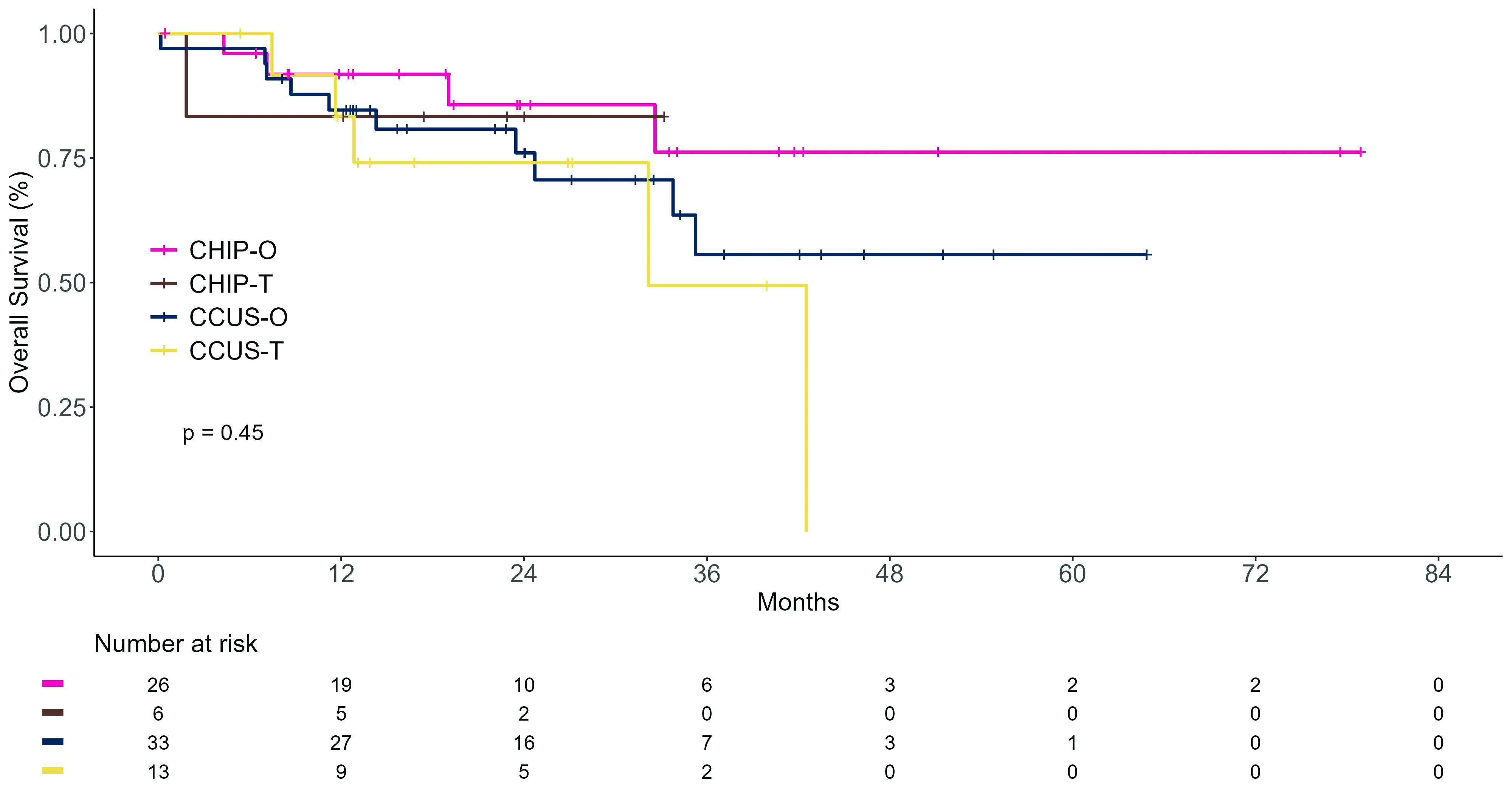

Supplement: Supplementary file 2 — Figure S1. [file CAM4-13-e7093-s003.tif]
